# Integrated bioinformatic analysis and machine learning strategies to identify new potential immune biomarkers for Alzheimer’s disease and their targeting prediction with geniposide

**DOI:** 10.1515/biol-2025-1215

**Published:** 2025-12-30

**Authors:** Fang He, Fang Fen Sha, Han Yi Hu, Hua Zan Zhang, Ruo Zhang

**Affiliations:** The Second Affiliated Hospital of Wenzhou Medical University, Wenzhou, 325027, China; Wenzhou Medical University, Wenzhou, 325035, China

**Keywords:** Alzheimer’s disease, machine learning, immune infiltration, geniposide, molecular dynamic simulation

## Abstract

To analyze the immune biomarkers, pathogenesis, level of immune infiltration, and anti-Alzheimer’s disease (AD) potential of geniposide in immune-related AD. The expression profiles of the GSE132903 dataset were downloaded from the gene expression omnibus (GEO) database to obtain differentially expressed genes (DEGs) in AD, while immune-related genes (IRGs) were obtained from the ImmPortal database, and these genes were intersected to obtain immune differential genes. These genes were intersected to obtain immune differential genes, which were subsequently enriched for further analysis. With the help of protein-protein interaction (PPI) network and cytoHubba analysis, the key immune differential genes were screened out, and the characteristic biomarkers were further identified and screened by the least absolute shrinkage and selection operator (LASSO) regression model and SVM-RFE algorithm. The (receiver operating characteristic) ROC curve was validated in the validation group of GSE5281 microarray and the area under the ROC curve value was used to evaluate the diagnostic and therapeutic values. The CIBERSORT algorithm was used to analyze the pattern of immune cell infiltration and the association between immune cells and characteristic biomarkers. Finally, geniposide was subjected to molecular docking and molecular dynamic simulations with core characterized genes to predict its anti-AD potential. In total, 345 DEGs were identified and 18 AD immune-related differential genes were identified by intersecting immune-related genes, which were involved in multiple signaling pathways, cellular components, molecular functions, and pathways. Five characterized genes were identified using integrated machine learning, including glial fibrillary acidic protein (GFAP), VGF Nerve Growth Factor Inducible (VGF), Neuropeptide Y (NPY), Cholecystokinin (CCK), and NFKB Inhibitor Alpha (NFKBIA). The ROC curve validation results were as expected. Immune cell infiltration analysis revealed that multiple immune cells were associated with the characterized genes. Molecular docking and molecular dynamic simulations showed good binding activity and stability between geniposide and the key characterized targets. Characteristic biomarkers of AD were screened using various methods, and the biological processes and signaling pathways related to AD were identified by enrichment analysis, which elucidated immune-related mechanisms. In addition, geniposide may have binding affinity for key target proteins involved in the pathogenesis of AD, suggesting its potential as a candidate worthy of further investigation. And this study provides a new approach to the pathogenesis and targeted therapy for AD.

## 1 Introduction

Alzheimer’s disease (AD), also known as primary degenerative dementia, often occurs in the elderly and is an irreversible and lethal degenerative disease of the central nervous system. It is often accompanied by progressive cognitive impairment, memory deficits, personality and behavioral changes, and other symptoms that seriously affect the quality of life of patients [1]. With an aging global population, the prevalence of Alzheimer’s disease will further increase. Relevant studies have reported that approximately 50 million patients with AD are present worldwide, and by 2050, the number of patients with AD will increase to 152 million, making it a major public health problem in the world [2]. AD has a complex pathogenesis, in which both abnormal accumulation of β-amyloid (Aβ) as well as Tau protein have been recognized as the hallmark pathological processes of AD. However, recent studies have found that it is the abnormal accumulation of Tau that is closely associated with brain atrophy, whereas Aβ seems to be a necessary, however, not a sufficient factor leading to AD [3]. However, the mechanisms underlying tau-mediated neurodegeneration remain elusive.

Increasing evidence suggests that AD is not only a protein disease but immune-inflammatory responses also play an important role in the development of AD, especially in immune cells, such as microglia, mast cells, and macrophages [4]. At different stages of AD, microglia polarize into pro-inflammatory M1 and neuroprotective M2 types. Once activated, microglia can enhance their phagocytosis to remove debris and toxins from the brain and precisely orchestrate a series of inflammatory responses to restore brain homeostasis [5], 6]. The balance of the two T cell subsets, T helper (Th) and regulatory T cells (Treg), is crucial for the maintenance of immune homeostasis in the body. Disruption of this balance triggers a drastic immune response *in vivo*, and changes in this balance may be closely related to the development of AD [7]. However, AD-related studies that systematically analyze core genes, assess differences in immune cell abundance, and determine correlations between immune genes and immune cells are still at a relatively early stage. Therefore, immune-related genes must be analyzed and screened to identify potential pathogenesis and diagnostic markers of AD, which is important for exploring effective preventive and curative measures for AD.

Although medications that improve cognitive symptoms can restore impaired functioning to some extent, they can also cause several adverse effects [8], 9]. Therefore, new treatments are urgently needed to prevent and slow the progression of AD. Clinical trials have shown that traditional Chinese medicine (TCM) has a positive effect on early prevention, and improves cognition and brain activity in patients with AD [10], 11]. A growing number of studies have suggested geniposide as a new therapeutic agent for AD [12], 13]. As a chemical component of iridoids in the heat-clearing and detoxifying traditional Chinese medicine Gardeniae Fructus, geniposide is the major substance in gardenia that exerts its pharmacological effects. However, the underlying mechanism requires further elucidation. Based on the above, this study obtained raw data from the gene expression omnibus (GEO) database and analyzed differential genes in brain tissue using the R language. Differential immune genes were first screened and analyzed for immune enrichment and the core genes were further explored using the STRING database. Machine learning was used to screen for immune-related markers and the core genes were analyzed by subject receiver operating characteristic curve (ROC) analysis to determine the diagnostic value of the key genes. Based on the CIBERSORT algorithm, we analyzed the immune cell infiltration pattern and the correlation between core genes and immune cells to explore the pathological mechanism, immune cell infiltration level, and potential therapeutic targets of AD. The core genes were then molecularly docked and molecular dynamics simulation with geniposide to provide ideas for study the molecular mechanisms underlying AD and therapeutic drugs.

## 2 Materials and methods

### 2.1 Downloading and processing of data

Using “Alzheimer’s disease” as the keyword, the GEO database (https://www.ncbi.nlm.nih.gov/geo/) was searched. Filter the dataset to include only those samples that meet the following criteria [1]: derived from human brain tissue [2], structured in a case-control design, and [3] have a sample size ≥20 per group to ensure representativeness. Two original datasets were obtained (GSE132903 and GSE5281). GSE132903 was based on the GPL10558 platform and includes 97 patients with AD and 98 normal (control) samples, whereas GSE5281 was based on the GPL570 platform and includes 87 patients with AD and 74 control samples. Subsequently, the probes in each dataset were converted to gene symbols based on the probe annotation files obtained from the GEO database. Expression profiles from the GSE5281 dataset were used as a training set and corrected for batch effects using the SVA algorithm for subsequent machine learning and immune correlation analyses. GSE5281 was used as an additional dataset for validation.

### 2.2 Differentially expressed gene (DEG) analysis

The metadata queue was analyzed for differences using the “limma” package in R software and DEGs were obtained by using the “FDR” processing method and setting the filter condition as log FC filter >1, corrected for *P* < 0.05. DEGs were displayed using ggplot2 and pheatmap for volcano and heat maps, respectively.

### 2.3 Differential immune-related gene (IRG) acquisition

Immune-related genes (IRGs) were downloaded from the ImmPort (https://www.immport.org/home) database, and the Venn Diagram package was used to identify common DEGs with DEGs, also known as intersecting differentially immune-related genes (DIRGs).

### 2.4 Enrichment analysis

To reveal the biological functions of DEGs, we used the “cluster Profiler” package in R to analyze the pathways of DEGs in Kyoto encyclopedia of genes and genomes (KEGG) and Gene ontology (GO) (*P* < 05), and selected the top 10 pathways of different entries of GO and the top 30 pathways of KEGG. The top 10 pathways with different entries of GO and the top 30 KEGG pathways were selected for visualization, and GO chord diagrams and KEGG enrichment histograms were drawn.

### 2.5 Protein-protein interaction net-work (PPI) network construction and Hub Gene identification

PPI networks contribute to the understanding of AD pathological pathways and interacting proteins, which may shed light on the pathogenesis of AD and its potential biomarkers. Cytoscape’s cytoHubba plugin supports the computation of a variety of network centrality parameters, including degree centrality (DC), betweenness centrality (BC). This provides a comprehensive perspective on complex network analyses. DIRGs were imported to the STRING website to construct human PPI networks, which were subsequently imported into Cytoscape software. The above parameters were set in the Cyto NCA plug-in for comprehensive computational analysis to obtain the top 10 ranked targets as Hub Genes.

### 2.6 Differential analysis of core gene expression

The core genes were similarly analyzed for differential expression according to the method described in item 2.2, and volcano plots and heat maps were constructed to better represent the expression of the core genes in the AD group versus the control group.

### 2.7 Correlation analysis of core genes

Gene expression correlation analysis is an analytical method used to study genomic data and determine the relationship between gene expression and specific physiological processes. It helps to identify differences in gene expression patterns between different diseases or other specific states to study the biological functions of genes. It also helps to identify and categorize classifications with similar gene expression patterns to study the pathogenesis of diseases, and thus provides intervention strategies for AD. Correlation analysis of core genes is performed with the help of the “corrr” and “ggplot2” packages of the R language.

### 2.8 Characterization biomarker screening

Two machine learning algorithms, namely, the least absolute shrinkage and selection operator (LASSO) and support vector machine (SVM), were used to predict the characteristic biomarkers [14]. First, Feature selection was carried out using LASSO regression, a method well-suited for high-dimensional data that shrinks coefficients and forces less contributive variables to zero. We implemented a binomial LASSO model (family = “binomial”) as our outcome was binary. The strength of the L1 penalty was controlled by the lambda parameter, the value of which was optimized through 10-fold cross-validation. The lambda value that yielded the minimum mean cross-validated deviance (lambda.min) was selected to fit the final model. The random seed was set to ensure the reproducibility of the cross-validation splits. All analyses were performed using the glmnet package (version 4.1.8) in R.

To identify a parsimonious set of predictive features, we employed Support Vector Machine Recursive Feature Elimination (SVM-RFE). The algorithm was configured with a linear kernel SVM to leverage the feature weights for ranking importance. A 10-fold cross-validation procedure was nested within the RFE process to robustly validate the predictive performance of each feature subset and to prevent overfitting. The feature elimination followed an optimized strategy: when the number of features exceeded 50, the least important half was removed per iteration (halve.above = 50). Otherwise, features were eliminated one-by-one. The final optimal feature set was defined as the subset that yielded the minimum average cross-validation error.

### 2.9 Diagnostic and therapeutic value determination of AD by characteristic biomarkers

To test the value of the feature genes obtained in 1.7, the sample data contained in the validation group were imported into the R software, and box plots were drawn using the “limma” and “ggpubr” packages to determine the value of the genes in the intersection of the LASSO and SVM-RFE algorithms. Using the “pROC” package in the R software, we plotted the receiver operating characteristic (ROC) curve using the same data, and determined the diagnostic validity of AD using the area under the curve (AUC) value. The diagnostic validity of AD was determined using AUC value. After selecting the best model, column line plots were constructed using the characterized genes and their expression levels in the control and AD groups, and decision and calibration curves were constructed to determine the accuracy of the column line plots.

### 2.10 Validation of external datasets

In addition, box-and-line plotting and calculation of the AUC for the data in the validation set were performed using the same method to validate the predictive accuracy, with *P* < 0.05 as the criterion for statistical significance.

### 2.11 Immune infiltration correlation analysis

CIBERSORT is an analytical tool based on support vector regression for assessing immune cell composition in the microenvironment. In this study, we applied the CIBERSORT algorithm to differentiate 22 human immune cell subpopulations, including myeloid, plasma, B, and T cells, based on the expression matrices of multiple biomarkers. The results were visualized using immune infiltration and box-line plots. In addition, we calculated the infiltration ratio of each type of immune cell and analyzed its correlation with gene expression levels using Pearson’s correlation coefficient, with *P*-value < 0.05 as the criterion for statistical significance.

### 2.12 Molecular docking and molecular dynamic simulations

As previously described in the literature [15], 16], to assess the interaction pattern between geniposide and the characterized targets, protein-ligand docking was performed using Autodock Vina 1.2.2. The molecular structures of the drugs were obtained from the PubChem Compound Database (https://pubchem.ncbi.nlm.nih.gov/). The 3D structures of the proteins were downloaded from the PDB (http://www.rcsb.org/). All protein and ligand files were first converted to PDBQT format by removing all water molecules and adding polar hydrogen atoms. Molecular docking was visualized using Autodock Vina 1.2.2 (http://autodock.scripps.edu/). To corroborate the credibility of our molecular docking findings for geniposide, we employed the same protocol to dock known anti-AD drugs, such as rivastigmine and donepezil [17], and conducted a comparative analysis of the binding affinity results.

Based on the molecular docking results, molecular dynamic simulations of the best complexes were performed using the Groningen Machine for Chemical Simulations (GROMACS) 5.0 software package and the CHARMM36 force field under molecular periodic boundary conditions [18]. Ligand topology files were generated using the CHARMM generic force field. The charge of the system was neutralized by the addition of ions. The energy was minimized using the steepest gradient method to eliminate close contact. Energy calculations and electrostatic and van der Waals interactions were performed using the particle-mesh Ewald (PME) method. The system was equilibrated for 50,000 steps in the NVT set and another 50,000 steps in the NPT set. Finally, a 100 ns molecular dynamic simulation was performed at 300 K with a time step of 2.0 fs, and the coordinates were saved in units per picosecond for analysis.

### 2.13 Statistical analysis

Statistical analyses in bioinformatic analysis were performed using R software (version 4.2.2).

## 3 Results

### 3.1 Screening for AD immune-related differential genes

**Figure 1:**
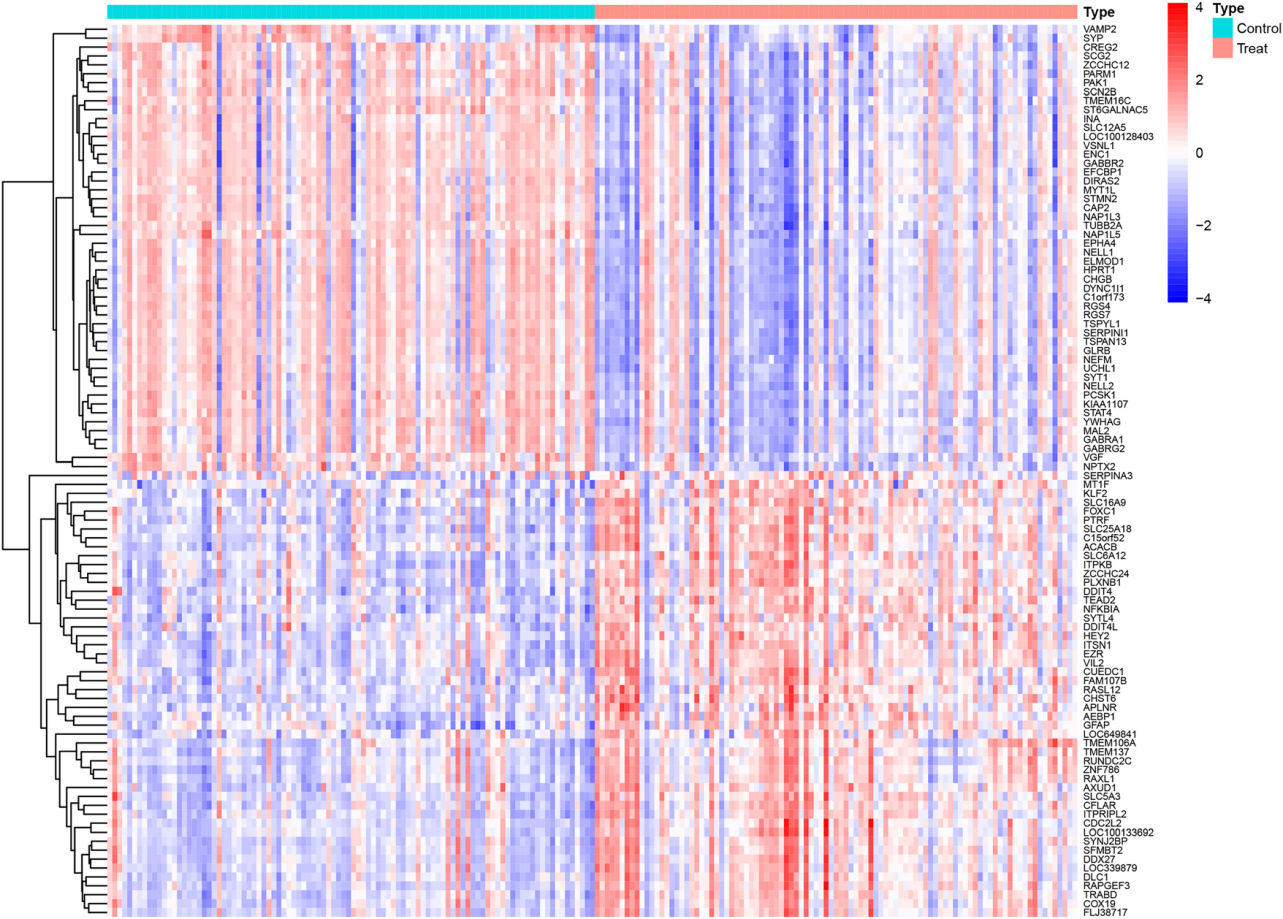
Differentially expressed genes analysis of healthy population and Alzheimer’s disease patients. A. Differential gene heatmap.

Through re-annotation and differential analysis in the R language, a total of 345 DEGs were identified after differential analysis between the control and AD groups, of which 132 were upregulated and 213 were downregulated in expression, as shown in Figure 1A. The volcano plot easily visualized the distribution of genes differentially expressed between the two samples and genes with large differences were distributed at both ends, as shown in Fig. S1A. Subsequently, we examined the intersection of differential genes with immune-related genes to obtain 18 AD immune-related differential genes, as shown in Fig. S1B.

### 3.2 Enrichment analysis

**Figure 2:**
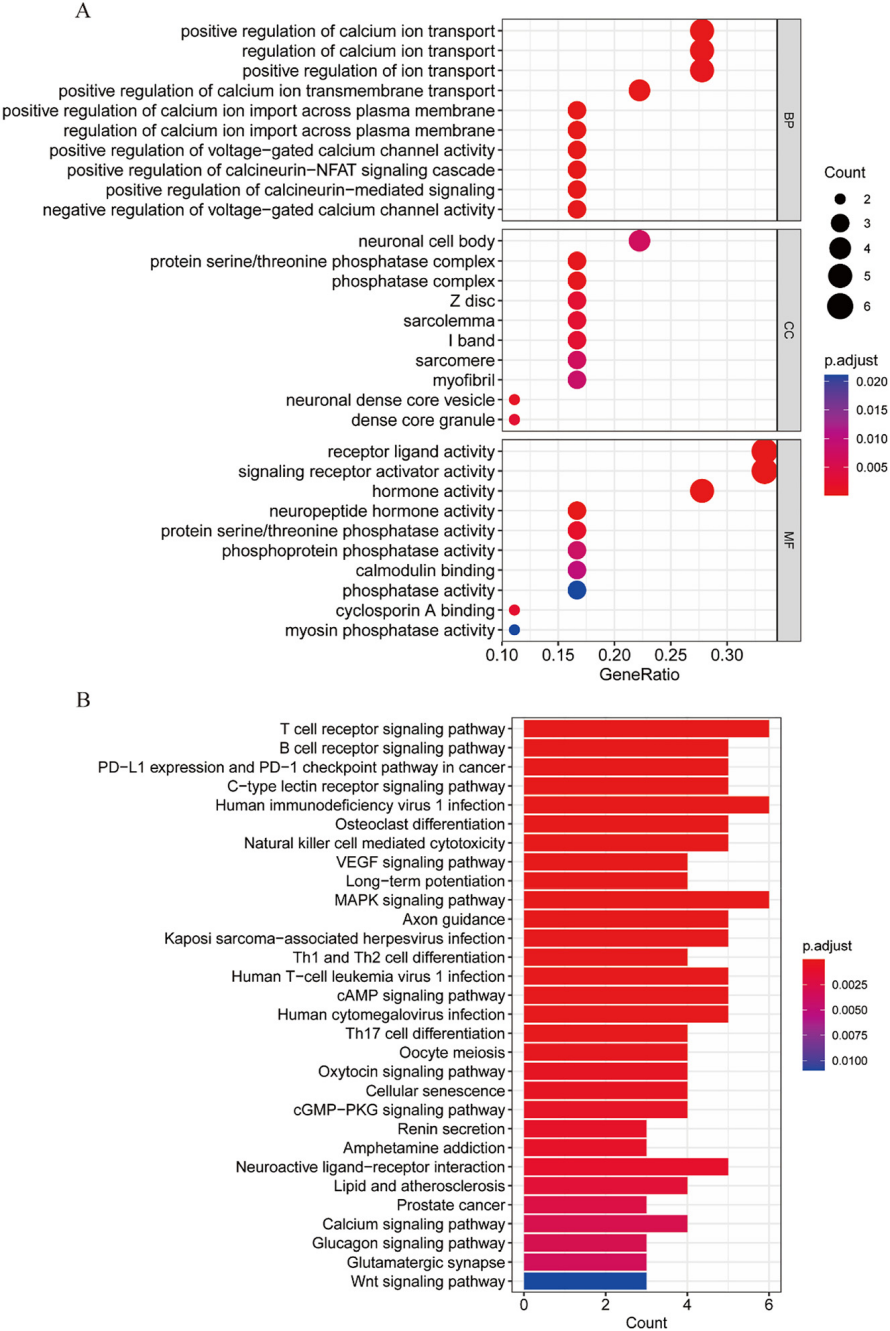
Immune differential gene enrichment analysis. A. GO functional enrichment analysis bubble plot. B. KEGG functional enrichment analysis histogram.

GO enrichment analysis of differentially expressed genes in patients with AD and normal controls revealed 154 biological processes, including the positive regulation of calcium ion transport, positive regulation of calcium ion import across the plasma membrane, and regulation of calcium ion import across the plasma membrane which included 25 cellular components, such as the protein serine/threonine phosphatase complex, phosphatase complex, and neuronal dense core vesicles. Fourteen molecular functions were identified, including hormone, neuropeptide hormone, and receptor ligand activity. The top 10 biological processes were screened from the three panels, and a GO functional analysis bubble diagram was constructed (Figure 2A). Further KEGG enrichment analysis of immune DEGs revealed that a total of 62 signaling pathways were in focus, among which T cell receptor signaling pathway, B cell receptor signaling pathway, PD-L1 expression and PD-1 checkpoint pathway in cancer were closely related to the development of AD, and KEGG pathway analysis bar graphs of the first 30 pathways would be constructed (Figure 2B).

### 3.3 PPI network and core gene screening and expression analysis

**Figure 3:**
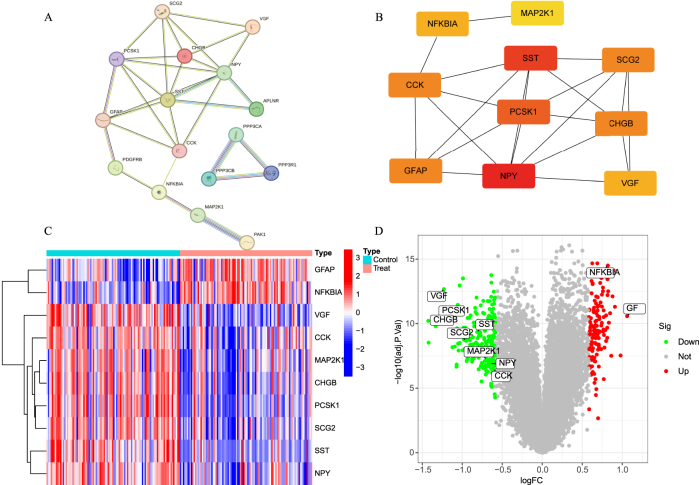
PPI network and core gene screening as well as expression analysis. A. Differential immunity gene PPI network map. B. Core differential immunity gene PPI network map. C. Core differential immunity gene heat map. D. Core differential immunity gene volcano map.

PPI analysis of the 18 intersecting targets was performed using the STRING database and the results showed that the network contained 18 nodes and 30 edges (Figure 3A). Based on the algorithm in CytoHubba, 10 core genes were screened and identified, as described in Figure 3B. Subsequently, we further analyzed the expression differences of these 10 core genes in the control and AD groups, as shown in Figure 3C, where red indicates an increase in the expression level and blue implies a decrease in the expression. In addition, the green color in the volcano in Figure 3D indicates a decrease in the expression level, and the red color indicates an increase in the expression level; the farther away from the center, the more the target should be concerned.

### 3.4 Correlation analysis of core genes

**Figure 4:**
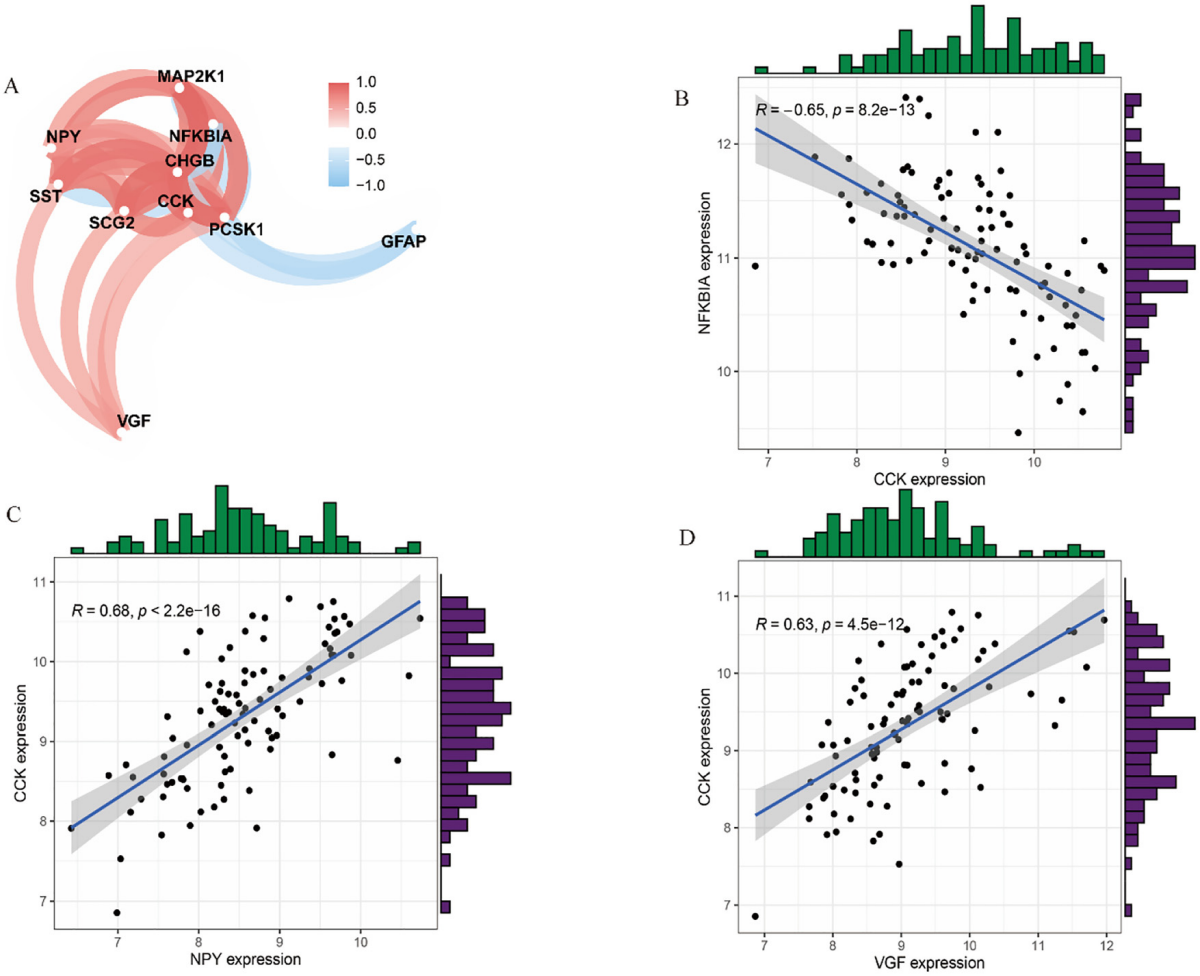
Correlation analysis of core genes. A. Correlation network diagram of core genes. B. Scatter histogram of correlation between NFKBIA and CCK. C. Scatter histogram of correlation between CCK and NPY. D. Scatter histogram of correlation between CCK and VGF.

Correlation analysis refers to the analysis of two or more variable elements that are correlated to measure the closeness of correlations between multiple variable factors. As shown in Figure 4A, the 10 genes were overall categorized into two groups, one of which was glial fibrillary acidic protein (GFAP) and NFKB inhibitor alpha (NFKBIA), which were positively correlated and negatively or uncorrelated with other genes. The other groups included VGF Nerve Growth Factor Inducible (VGF), SST, neuropeptide Y (NPY), MAP2K1, CHGB, cholecystokinin (CCK), PCSK1, and CCK, all of which were positively correlated. The significance of the correlations and correlation coefficients were further analyzed (Figure 4B–D). NFKBIA was significantly negatively correlated with CCK and the correlation coefficient *R* = −0.65, whereas CCK showed a significant positive correlation with NPY, with a correlation coefficient *R* = 0.68. Additionally, a significant positive correlation was observed between CCK and VGF, with a correlation coefficient of *R* = 0.63. The remaining significant correlations and coefficients are shown in Supplementary Fig. S2.

### 3.5 Characteristic gene screening

**Figure 5:**
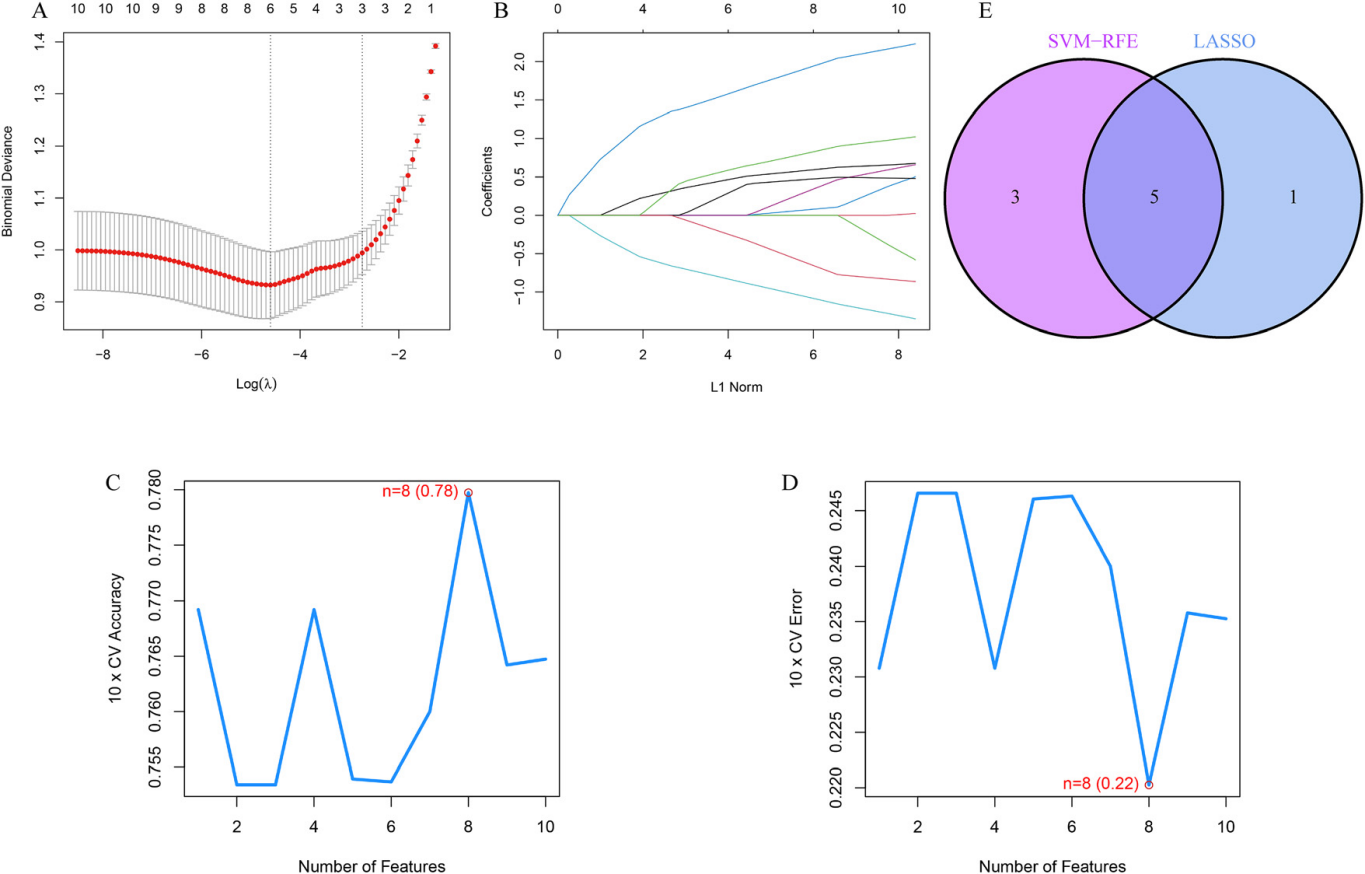
Two machine learning methods to screen potential markers for AD. A–B. Results of LASSO regression analysis. B–C. Results of SVM-RFE analysis. E. Intersection of two machine learning methods to obtain venn plot of characterized genes.

The core genes were narrowed down using the LASSO algorithm and six variables were identified as diagnostic biomarkers of AD pathogenesis, as shown in Figure 5A and B. The SVM-RFE algorithm was used to identify a subset of eight features in the core immune genes, as shown in Figure 5C and D. Five overlapping characterized genes (GFAP, VGF, NPY, CCK, and NFKBIA) were selected by the two algorithms, as shown in Figure 5E.

### 3.6 Diagnostic and therapeutic value determination of AD by characteristic biomarkers

**Figure 6:**
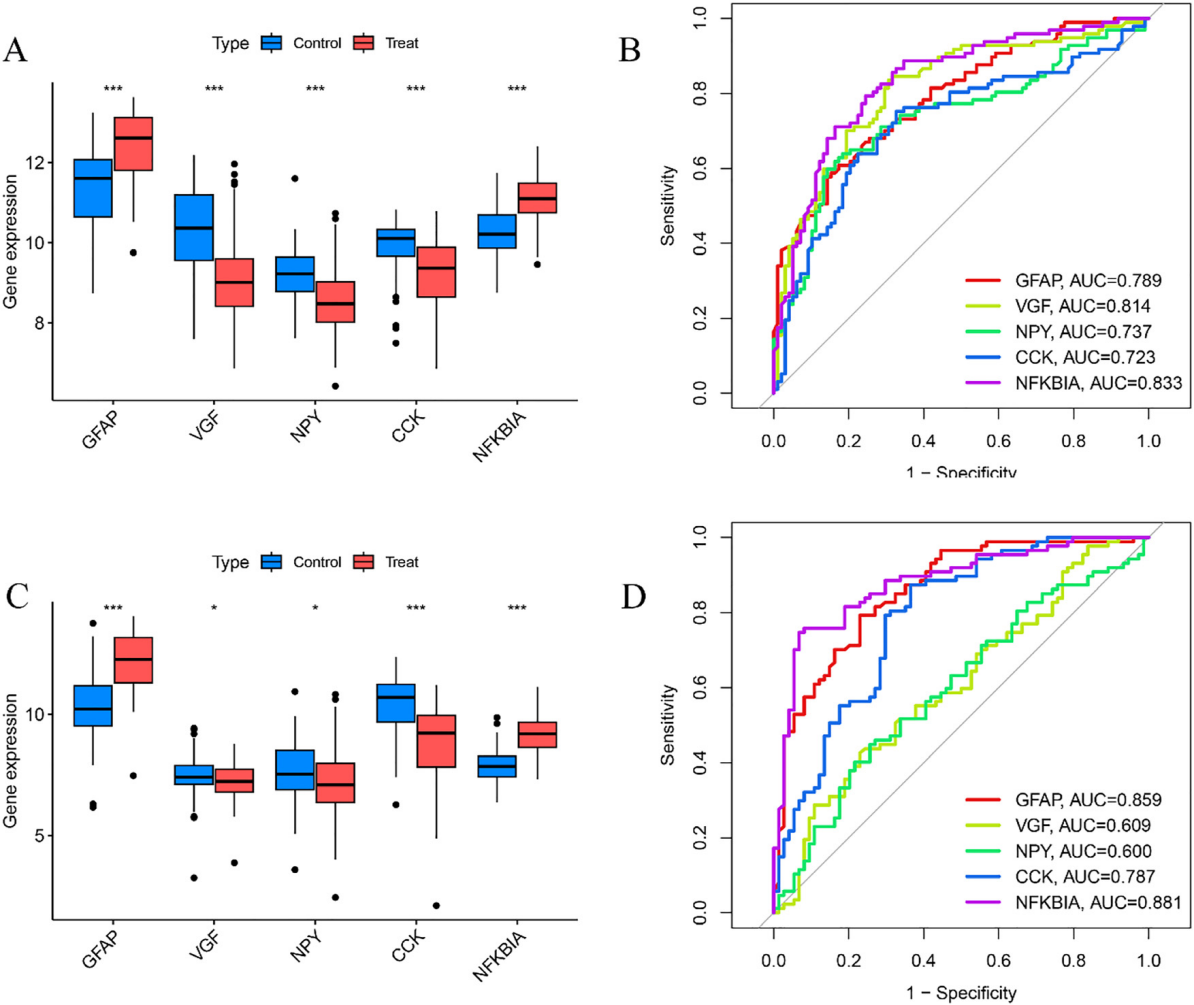
Diagnostic and therapeutic value determination of AD by characteristic biomarkers. A. Differential expression boxplots of the five characterized genes in the training set. B. Subject work characteristic curves for the five characterized genes in the training set. C. Differential expression boxplots of the five characterized genes in the validation set. D. Subject work characteristic curves for the five characterized genes in the validation set.

The expression levels of the characterized genes in AD were analyzed using the test set data, which showed that the expression of GFAP and NFKBIA was significantly elevated in the AD group with statistically significant differences (*P* < 0.05), whereas the expression of VGF, NPY, and CCK was significantly reduced in the AD group (*P* < 0.05) (Figure 6A). The ROC curve analysis of the characterized genes in the training set showed that the AUC values were greater than 0.7 (Figure 6B). In the validation set data, the expression trends of GFAP, VGF, NPY, CCK, and NFKBIA were similar to those in the training set and were differentiated (*P* < 0.05) (Figure 6C). In addition, ROC curve analysis was performed in the validation set, and the results showed that the AUC of GFAP, VGF, NPY, CCK, and NFKBIA were greater than 0.6 (Figure 6D). Individual scoring scales were obtained for these five characterized genes and treatment sensitivity was determined by calculating the sum of the characterized gene expression scores to predict the risk rate of the characterized genes in the development of AD (Fig. S3A). High prediction accuracy can be determined from the distance between the solid and dashed lines in the calibration curve (Fig. S3B).

### 3.7 Analysis of immune infiltration and correlation between key characterized genes and immune cells

**Figure 7:**
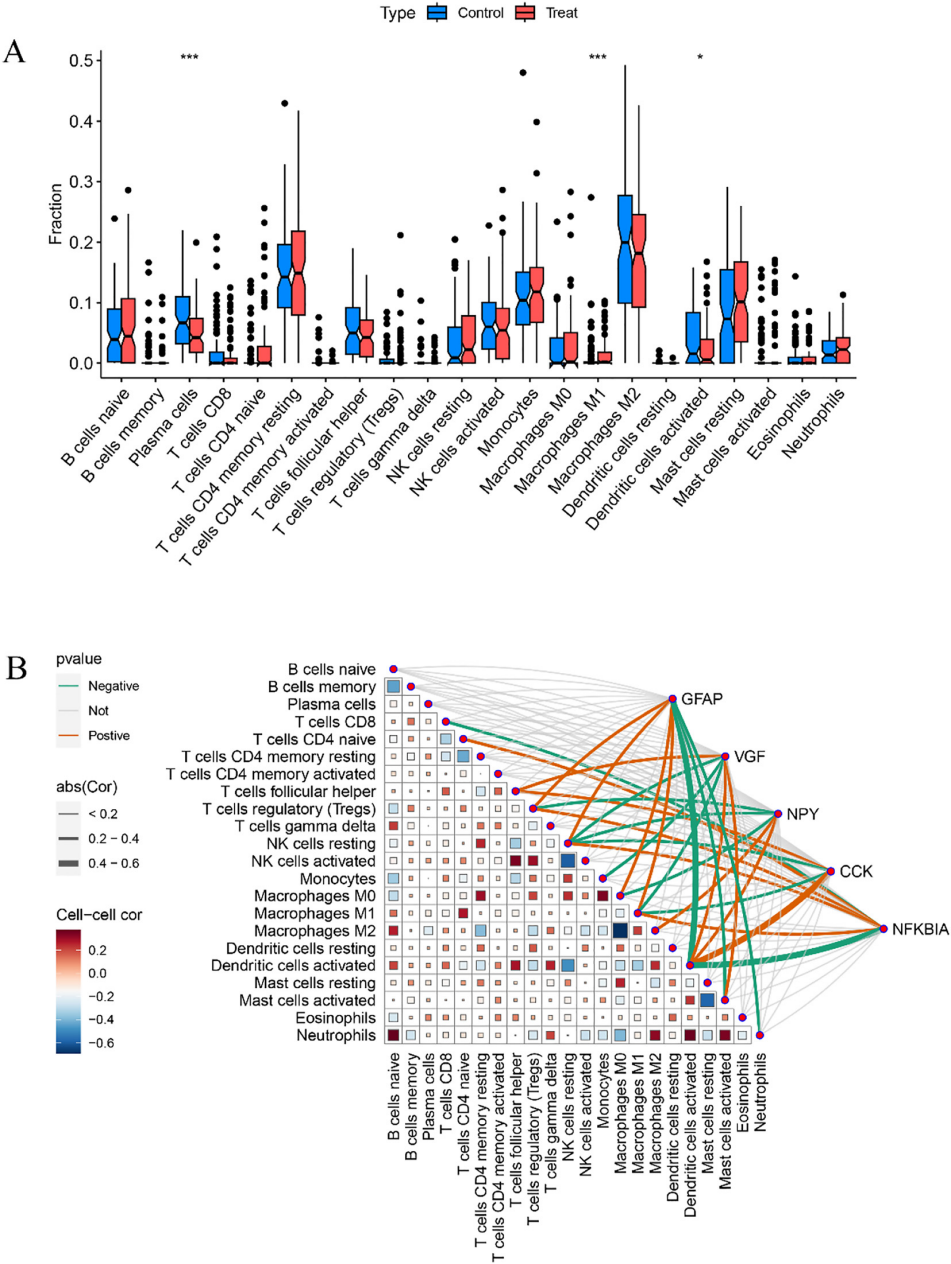
Analysis of immune infiltration and correlation between key characterized genes and immune cells. A. Box line plot of differences in immune cell scores. B. Plot of correlation analysis of key characterized genes with immune cells.

An immune cell infiltration analysis was performed to determine the types of immune cells expressed and their contents in each sample, and the results are shown in Fig. S4. The differences in immune cell infiltration between the AD and control groups were subsequently analyzed and activated plasma cells and dendritic cells (DCs) were significantly under expressed in the AD group. In addition, Macrophages M1 were highly abundant in the AD group (Figure 7A). Immune cell correlation analysis showed that blue indicates the presence of a negative correlation, and red represents a positive correlation. These five key genes were further analyzed for their correlation with immune cells, where the red line represents a positive correlation and the green line represents a negative correlation; the thicker the line segment, the more significant it is. Taking GAFP and VGF as examples, GFAP is positively correlated with regulatory T cells (Tregs) and resting NK cells, and negatively correlated with dendritic cell activation, mast cell activation, and neutrophils. VGF positively correlated with follicular helper T cells, activated dendritic cells, and activated mast cells, and negatively correlated with resting NK cells, Monocytes and Macrophages M1(Figure 7B).

### 3.8 Molecular docking and molecular dynamic results

**Figure 8:**
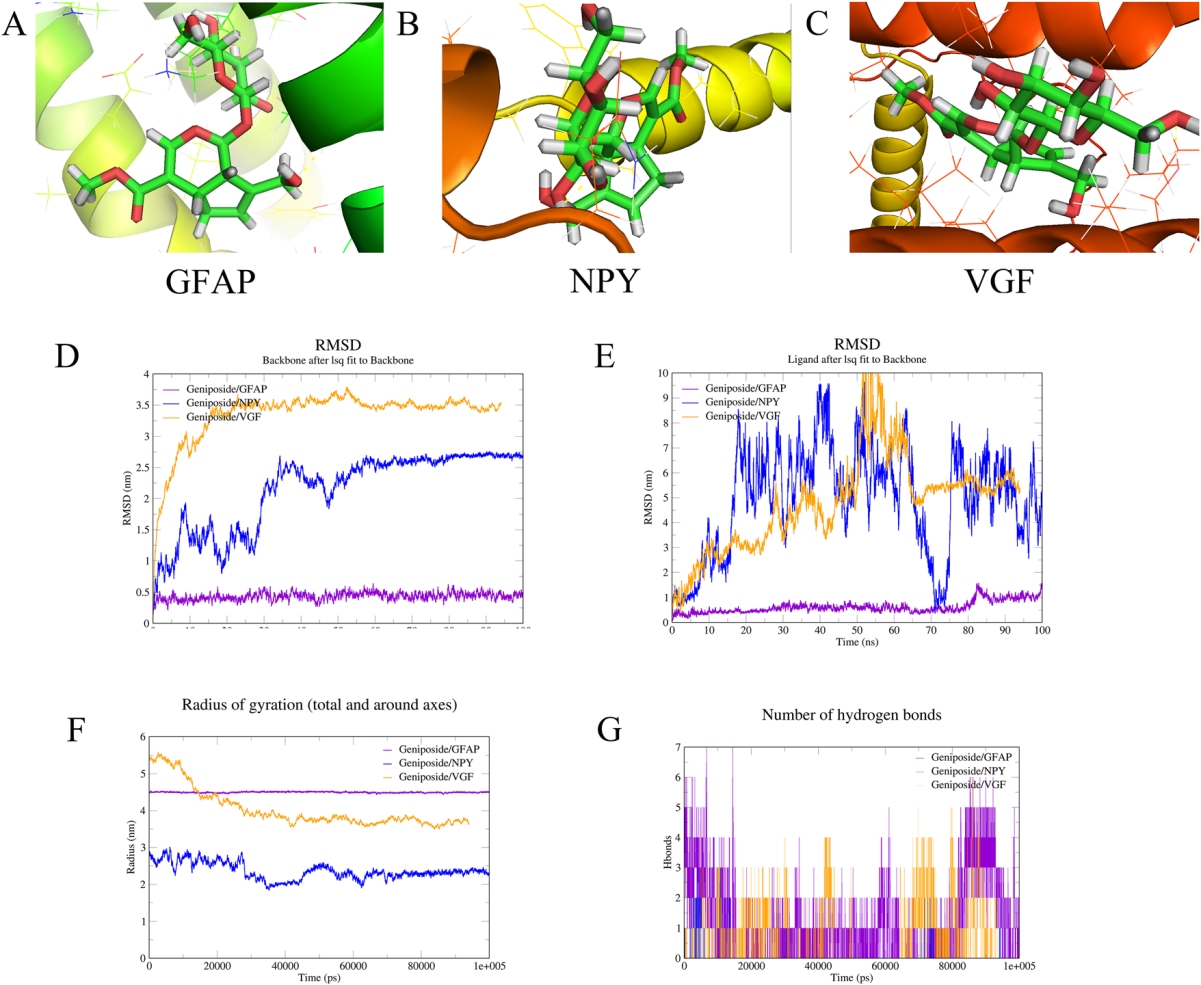
Molecular docking and molecular dynamics results. A. Visualization of molecular docking of Geniposide with GFAP; B. Visualization of molecular docking of Geniposide with NPY; C. Visualization of molecular docking of Geniposide with VGF; D. RMSD values of three proteins; E. RMSD values of three proteins after binding to Geniposide; F. Radius of gyration of three proteins binding to Geniposide; G. Number of hydrogen bonding of three proteins binding to Geniposide.

Geniposide was molecularly docked with GFAP, VGF, NPY, CCK, and NFKBIA. The AutoDock software was used to calculate the lowest binding energy of the geniposide and anti-AD drugs (rivastigmine and donepezil) to the targets and the specific results are shown in Table S1. It is generally believed that binding energy less than −5 kcal mol^−1^ indicates good binding activity between ligand and receptor, whereas when it is less than −7 kcal mol^−1^ indicates strong binding activity between ligand and receptor. The results showed that majority of binding energies after docking were less than −6 kcal mol^−1^, and the binding energy of geniposide to each target is between rivastigmine and donepezil, which indicated a good binding activity and stability between geniposide and the key characterized targets. The top three targets in terms of binding energy are presented with geniposide molecular docking results, as shown in Figure 8A–C. Subsequently, the top-three ranked targets were subjected to molecular dynamic simulations to verify the reliability of the molecular docking results.

Figure 8D shows the raw root mean square deviation of the three characterized proteins. Molecular dynamic simulations of geniposide with GFAP, VGF, and NPY were performed at 100 ns to determine the conformational stability of their complexes, and the results are shown in Figure 8. We considered GFAP as an example, the fluctuation of root-mean-square deviation (RMSD) curves of geniposide with GFAP was relatively stable from 0 to 100 ns (Figure 8E), and the root mean square fluctuation (RMSF) values were relatively small (see Supplementary Fig. S5), indicating that the binding of geniposide to GFAP has high stability. Meanwhile, the radius of gyration (Rg) value of geniposide to GFAP within 100 ns was extremely low (Figure 8F), and the maximum number of hydrogen bonds was seven (Figure 8G), suggesting that geniposide and GFAP may be through the hydrogen bonding tightly bound to GFAP. The Gibbs energy spectrum showed that the binding conformation of geniposide to GFAP was in a relatively stable state when the Rg value was 4.50–4.60 and the RMSD value was 0.40–0.60 (see Supplementary Fig. S6).

## 4 Discussion

AD is a neurodegenerative disease characterized by cognitive decline and memory loss; however, its pathogenesis remains unclear. Immune mechanisms are closely associated with AD development. However, few reports are available on potential markers of immune-related genes and corresponding therapeutic Chinese medicines in the field of AD research. Therefore, in this study, we analyzed the diagnostic markers, infiltration patterns, and biological pathways of immune-related genes in AD and predicted the potential of geniposide against AD using bioinformatics, machine learning, and molecular docking methods.

Machine learning has been successfully applied in multiple fields, including but not limited to the mining of feature genes [19], 20]. In this study, we identified five immunomarkers, GFAP, VGF, NPY, CCK, and NFKBIA, that were differentially expressed in patients with AD and controls and were closely related to AD development. Therefore, an in-depth exploration of their functions and related regulatory mechanisms can help enhance our understanding of the immune-related mechanisms of AD and provide new directions for the treatment of AD. GFAP is the backbone protein of astrocytes, which fills the cytosol and protrusions of astrocytes, and is recognized as a characteristic marker of astrocytes. Various types of injuries to the central nervous system can cause astrocytic responses characterized by increased GFAP levels, which is an early indicator of brain injury [21], 22]. Clinical studies have shown that patients with high levels of GFAP in their blood undergo more severe development of pathological tau proteins in the brain, which can be used as a basis for determining AD prior to clinical confirmation of the diagnosis, which is consistent with the findings of this study [23].

The neuroendocrine regulatory peptide VGF, nerve growth factor inducible, is produced by specific neuronal and neuroendocrine cells and is widely found in the central and peripheral nervous systems, participating in various neuroendocrine regulatory processes [24]. To evaluate VGF as a biomarker for the clinical diagnosis of AD, researchers collected cerebrospinal fluid from patients with AD and measured changes in VGF expression levels. The results of these studies showed that VGF levels were reduced in patients with AD compared to controls, and decreased further as the disease progressed [25], 26]. It was demonstrated *in vivo* in animals that VGF overexpression partially rescued β-amyloid-mediated neurodegenerative deficits and cognitive deficits in mice [27]. VGF hydrolyzed by neuroendocrine enzymes generates various derivative peptides, such as the C-terminal-derived derivative peptides TLQP-62 and TLQP-21, which can activate neuronal cell-surface receptors and regulate the formation of long-term hippocampal memories, as well as prevent immune-mediated memory deficits and depressive-like and anxiety-like behaviors in mice [28]. In addition, *in vivo* and *in vitro* data demonstrated that TLQP-21 administration to 5xFAD mice reduced amyloid pathology and microglial proliferation, as demonstrated in human microglial experiments [29]. Overall, these data highlighted the role of VGF as a potential biomarker and therapeutic target for AD.

Neuropeptide Y (NPY) is a 36 amino acid neuropeptide that is widely distributed in the mammalian central and peripheral nervous systems and is one of the most abundant neuropeptides. It acts as a natural ligand for G-protein-coupled receptors and plays a neuroprotective role in the prevention of neurodegenerative diseases [30]. For example, in AD caused by extracellular amyloid beta (Aβ) deposition to form Aβ plaques and intracellular tau protein hyperphosphorylation to form neuroprogenitor fibril tangles, Aβ creates Ca^2+^-permeable pores in neuronal cell membranes, through which excess Ca^2+^ influxes and a series of neurotoxic responses ensues. NPY reduces Aβ neurotoxicity by inhibiting the opening of voltage-gated calcium channels and lowering intracellular calcium concentrations. NPY binds to Y1 receptors to exert a reduction in microglial cell activity, inhibit their migration and phagocytosis, and reduce the release of IL-1β and TNF-α, which in turn reduces AD neuroinflammation and protects neurons [31]. Neuronal loss is a typical pathological change in AD, whereas NPY induces neural stem cell proliferation, differentiation, and nerve regeneration. Injection of NPY into the lateral ventricles of adult C57BL/6 rats revealed that NPY stimulated neuronal cell proliferation in the subventricular zone of the lateral ventricles and the subgranular zone of the dentate gyrus through the Y1 receptor, and induced progenitor cells to migrate from the subventricular zone of the lateral ventricles to the olfactory bulb and striatum for differentiation. High concentrations of NPY also promote the upregulation of the expression levels of brain-derived neurotrophic and nerve growth factors, which provide nutritive support for neoplastic cells and induce nerve regeneration [32].

Cholecystokinin (CCK) is a 33-amino acid peptide hormone found in the nervous system and gut. Numerous studies have shown that CCK plays an important role in the regulation of learning and memory by affecting the functions of neurons and glial cells. Therefore, CCK may be a useful marker of cognitive and neurological integrity in individuals with normal cognition, mild cognitive impairment, or Alzheimer’s disease (AD). Using data from the ADNI, one study examined whether CSF CCK levels correlate with onset and severity across the AD spectrum, and the results of this analysis suggested that CCK levels may reflect compensatory protection as AD pathology progresses [33]. Once in the brain, CCK can exert both direct and indirect protective effects, such as the vagus nerve-mediated upregulation of BDNF and NGF expression in the brain [34]. CCK also reduced the amyloid plaque load in the brains of APP/PS1 mice, whereas the neuroprotective effects of CCK analogs were attenuated by CCKB receptor antagonists and targeted knockdown of the CCKB receptor (CCKBR). These results suggest that CCK and its analogs ameliorate AD [35], 36].NFKBIA is a transcription factor that forms a complex with nuclear factor-κB (NF-κB) and prevents NF-κB from entering the nucleus of the cell, which in turn affects biological processes, such as immune response, apoptosis, and proliferation, and mediates the progression of AD. In addition, a recent study showed that the NF-κB signaling pathway is enriched in longevity-related genes, and elevated NFKBIA inhibits the expression of the NF-κB signaling pathway, leading to cognitive decline, dementia, and AD [37].

The CIBERSORT algorithm and its standard LM22 feature matrix are mainly used to analyze immune cells in peripheral blood and solid tumors. However, this algorithm has been extensively reported in the immune correlation analysis of AD [38], [39], [40]. It should be acknowledged that the brain has a unique immune environment dominated by resident microglia, which differ from peripheral macrophages in terms of individual development and function. Therefore, using this tool to deconvolve a large amount of brain tissue transcriptome data may not fully capture the complexity and specific activation states of neuroimmune cells. Although our results showed significant differences in estimated immune cell scores between groups, consistent with known AD pathophysiology, these findings should be interpreted as relative estimates rather than absolute measurements. Future research will benefit from the development and application of brain specific cell feature matrices extracted from single-cell RNA sequencing data [41], [42], [43] to achieve higher resolution and accuracy in characterizing the neuroimmune response of AD.

Molecular docking and molecular dynamic simulations showed that geniposide docked stably with five characterized biomarkers with good binding abilities. Previous studies have shown that geniposide may protect HT22 cells from Aβ-triggered neurodegenerative injury by activating mitochondrial autophagy [44]. Geniposide attenuates cognitive deficits and brain damage in APP/PS1 mice, and its neuroprotective effects may be mediated by modulation of the mTOR signaling pathway [45]. In addition, similar studies have reported that the beneficial effects of geniposide on the neuropathic damage and cognitive deficits characteristic of AD are based on the down-regulation of mTOR signaling, which leads to autophagy and lysosomal clearance of Aβ protofibrils [46]. The anti-AD effect of geniposide is still mainly focused on autophagy; however, this experiment correlates the pathogenesis of AD with the potential efficacy of geniposide from an immune perspective, expanding the potential mechanisms and targets of geniposide against AD based on a previous study. In short, geniposide may have binding affinity for key target proteins involved in the pathogenesis of AD, suggesting its potential as a candidate worthy of further investigation. However, this study was based on a biological database and simulation calculations; therefore, the results still need to be further verified by *in vitro* and *in vivo* experiments and clinical observations.

## 5 Conclusions

This study analyzed the AD immune-related markers and infiltration patterns using different techniques and obtained the AD-related biological processes and signaling pathways by key gene enrichment analysis, which provided a certain basis for immune regulation of AD and further clarified the pathogenesis of AD. Finally, molecular docking and molecular dynamic simulation analyses of the key immunological genes with geniposide were carried out, providing a reference basis for the prevention and treatment of AD by geniposide.

## Supplementary Material

Supplementary Material

Supplementary Material

Supplementary Material

Supplementary Material

Supplementary Material

Supplementary Material

Supplementary Material

Supplementary Material

Supplementary Material

Supplementary Material

Supplementary Material

Supplementary Material

Supplementary Material

Supplementary Material

Supplementary Material

Supplementary Material

Supplementary Material

Supplementary Material

Supplementary Material

Supplementary Material

Supplementary Material

Supplementary Material

Supplementary Material

Supplementary Material

Supplementary Material
