# Supplementary material for: Integrated bioinformatic analysis and machine learning strategies to identify new potential immune biomarkers for Alzheimer’s disease and their targeting prediction with geniposide
Source: Open Life Sci. 2025 Dec 30;20(1):20251215. doi: 10.1515/biol-2025-1215 (PMC13011607; doi:10.1515/biol-2025-1215)
Supplement: Supplementary file 25 — Supplementary Material [file j_biol-2025-1215_suppl_025.docx]

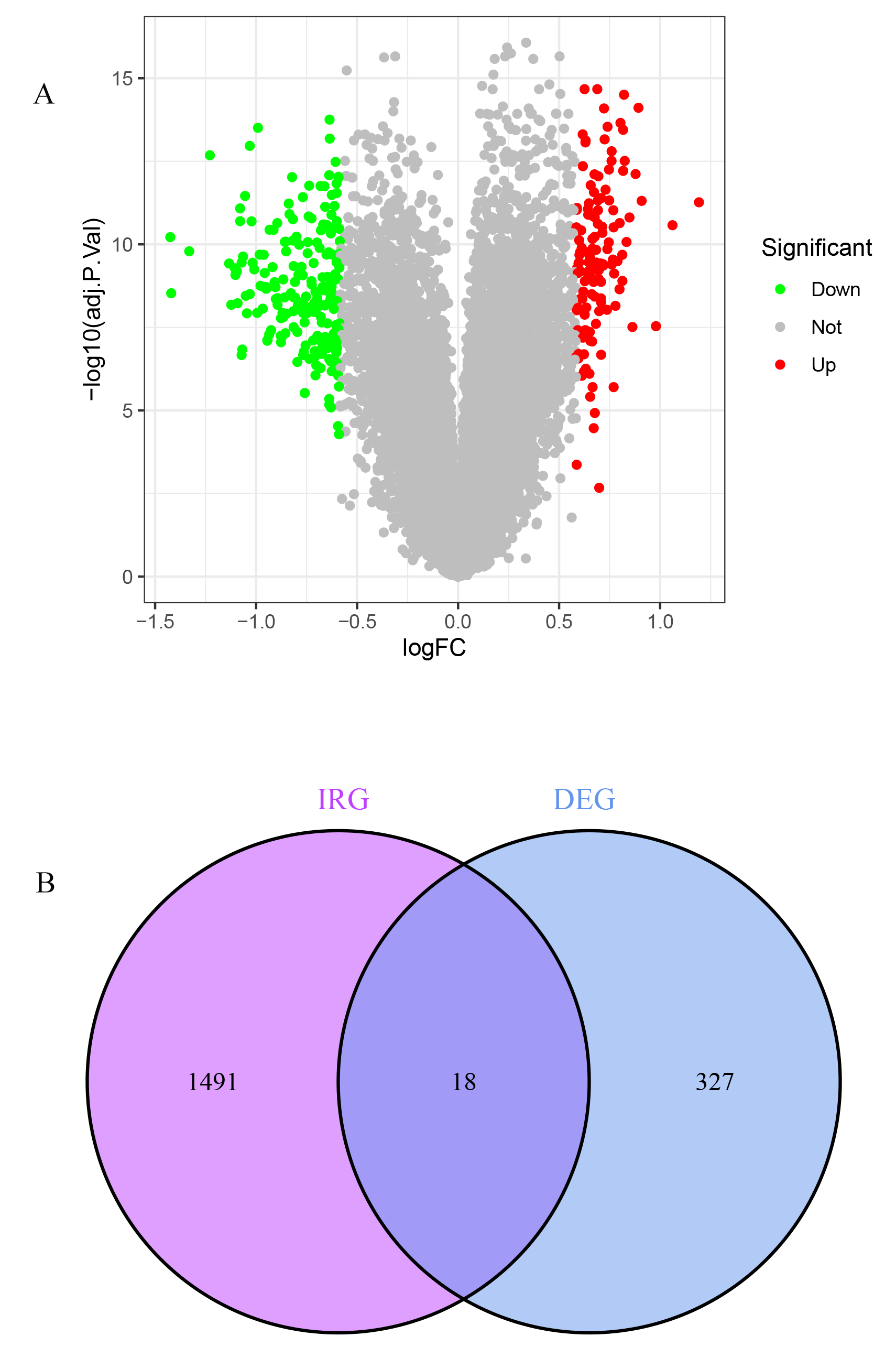


Figure S1. (A) Differential gene volcano map. (B) Venn diagram of intersection between differentially expressed genes and immune related genes. The pink part represents the number of immune related genes, the light blue part represents the number of differentially expressed genes, and the intersection part represents immune related differentially expressed genes.


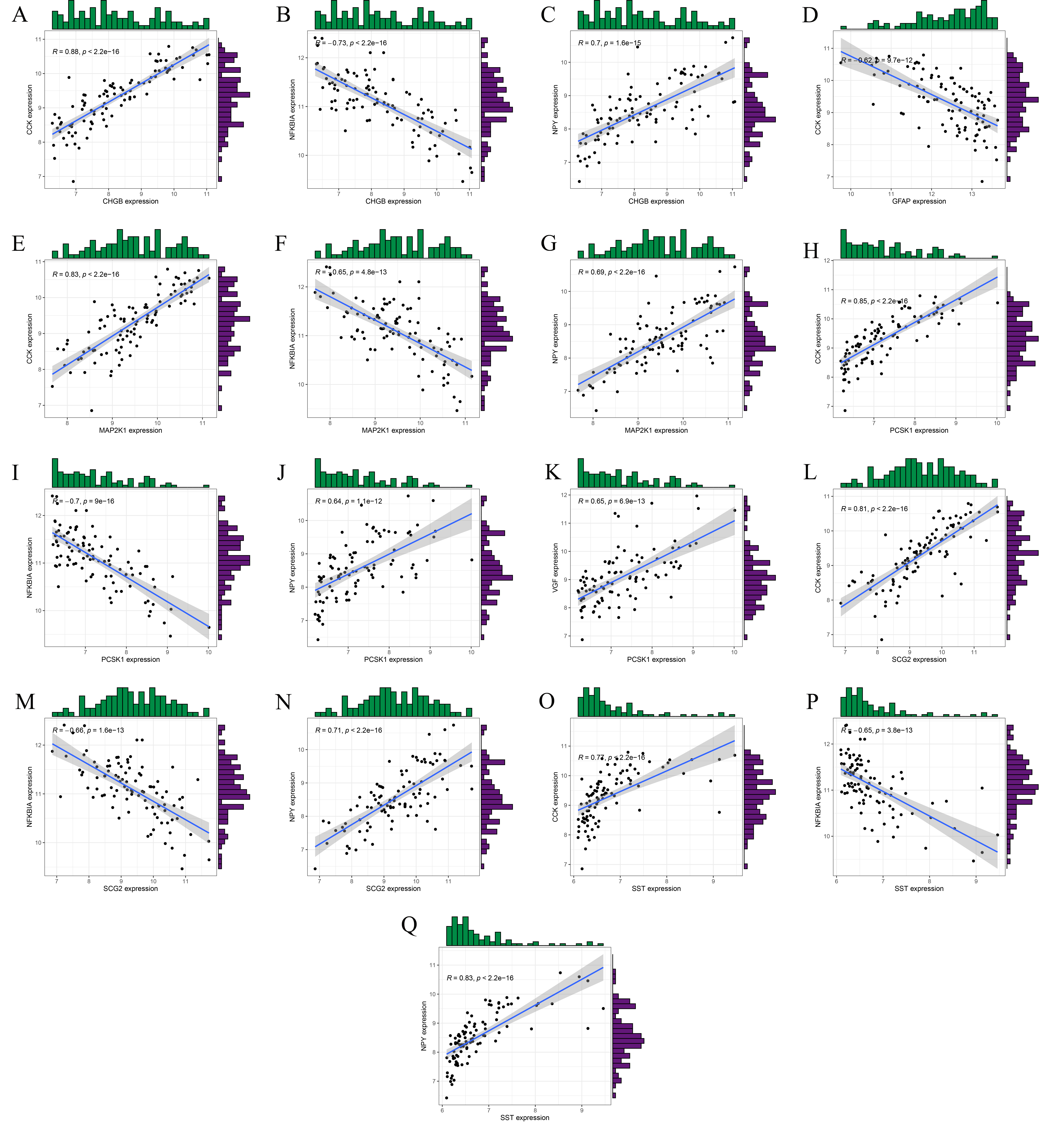


Figure S2. Correlation analysis of core genes.


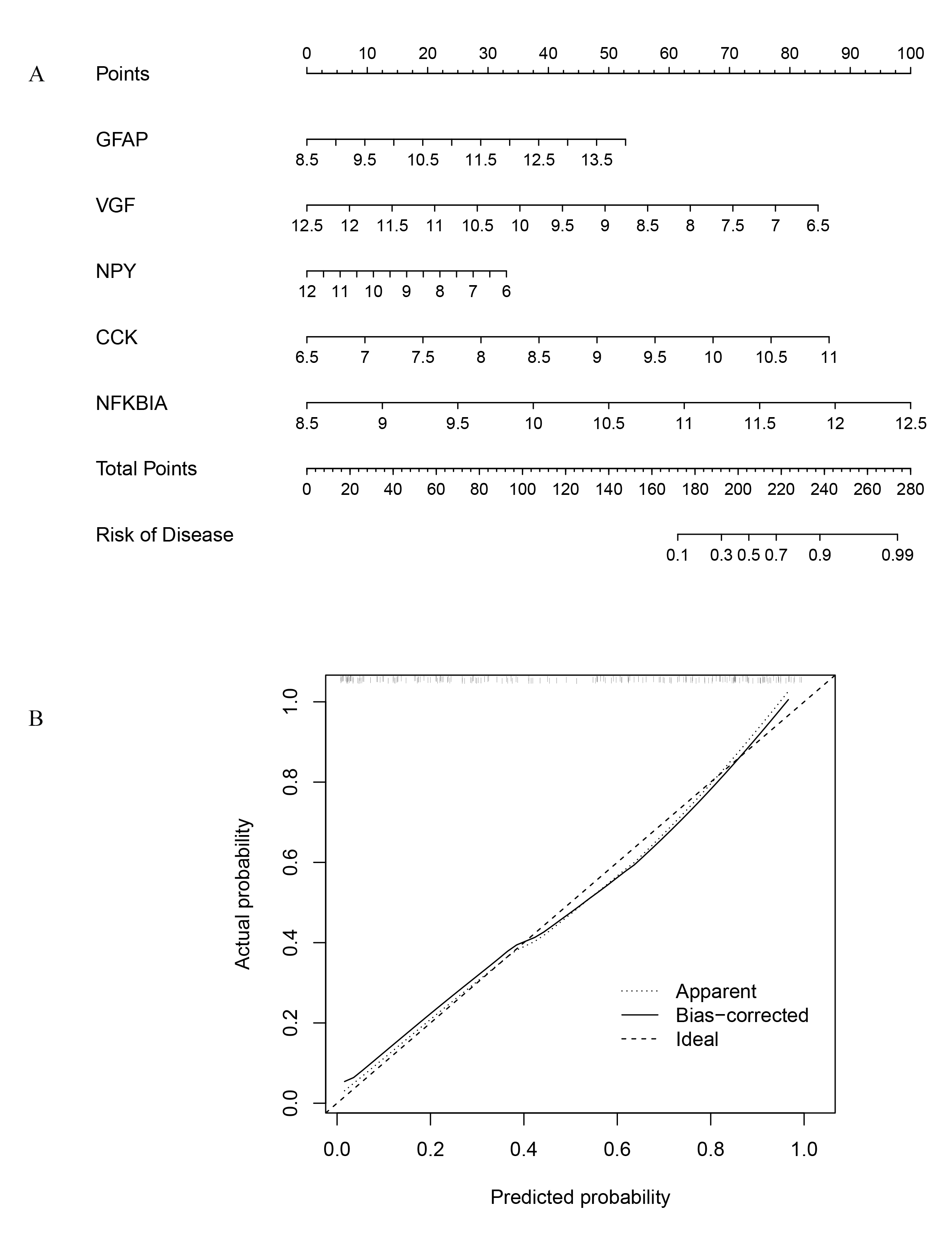


Figure S3. (A) Characteristic gene column line plots. (B) Correction curves for the characteristic gene column line plots.


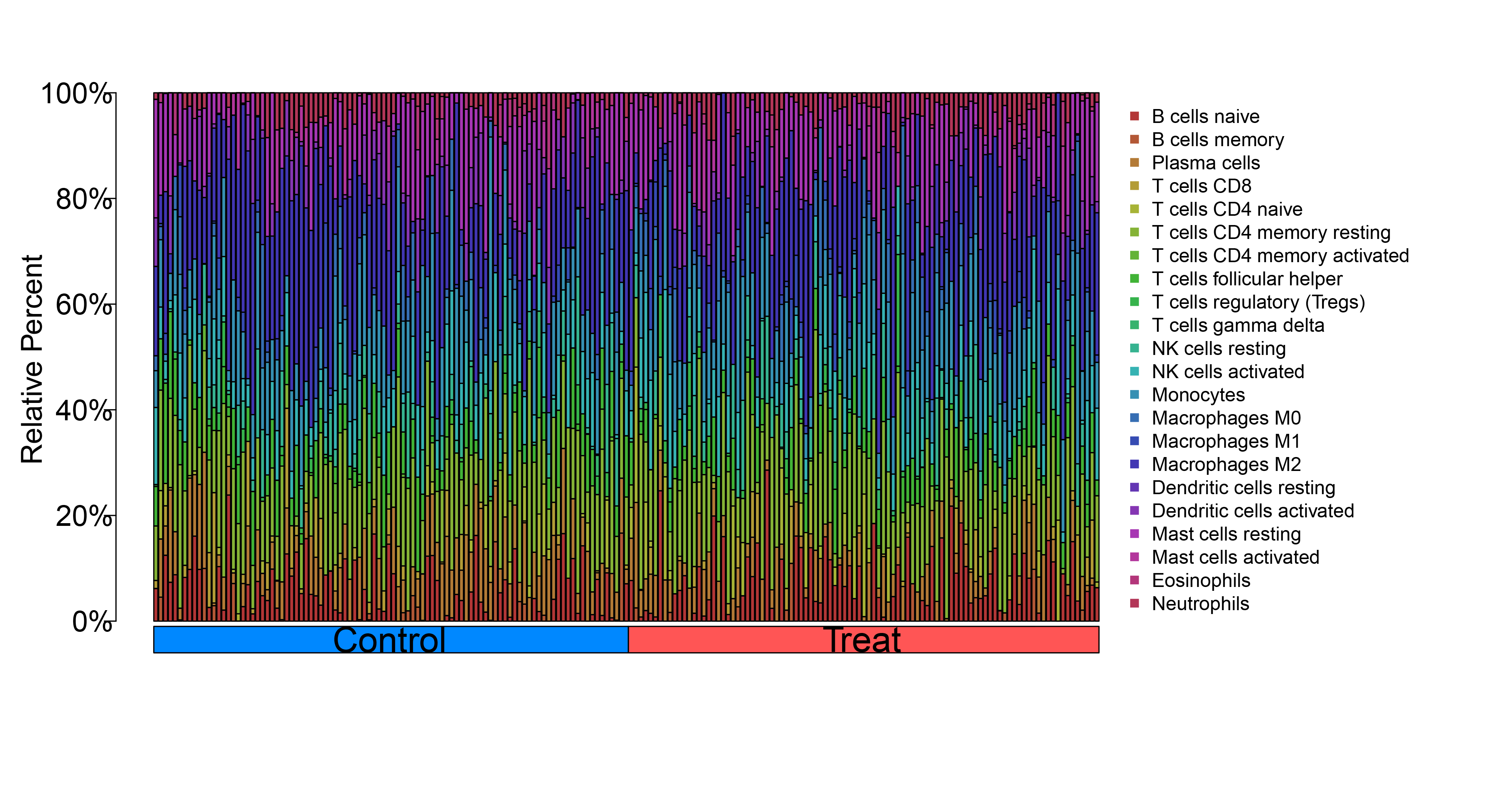


Figure S4. Plot of relative content of different immune cells.

Table S1 Molecular docking results of Geniposide with five characterized markers. (kcal/mol )

|  | GFAP | NPY | VGF | CCK | NFKBIA |
| --- | --- | --- | --- | --- | --- |
| Geniposide | -7.0 | -7.6 | -8.6 | -6.0 | -5.9 |
| Rivastigmine | -5.5 | -6.3 | -6.4 | -6.3 | -4.6 |
| Donepezil | -7.3 | -9.1 | -9 | -9.1 | -6.7 |


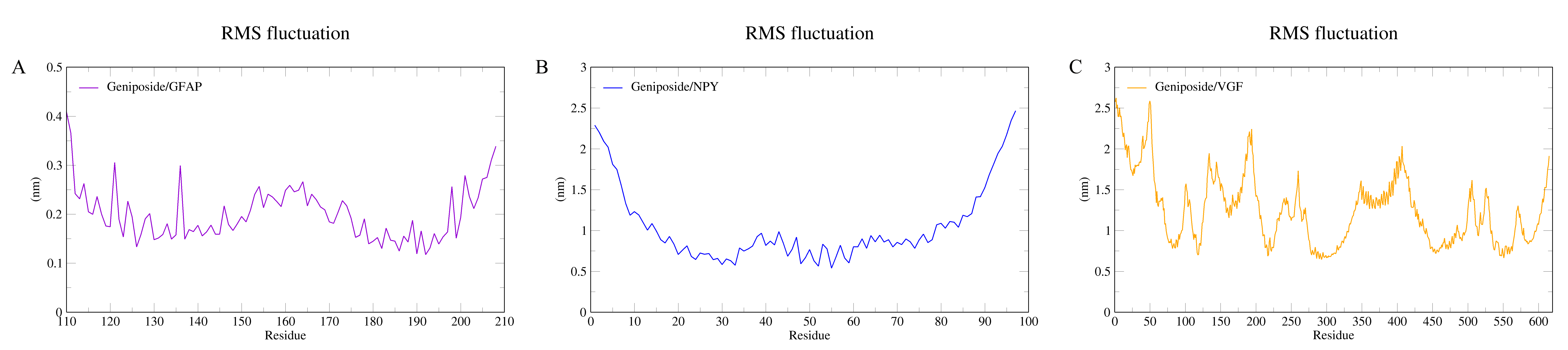


Figure S5. Molecular dynamics simulation results. A. RMSF of GFAP with Geniposide; B. NPY of GFAP with Geniposide; C. RMSF of VGF with Geniposide


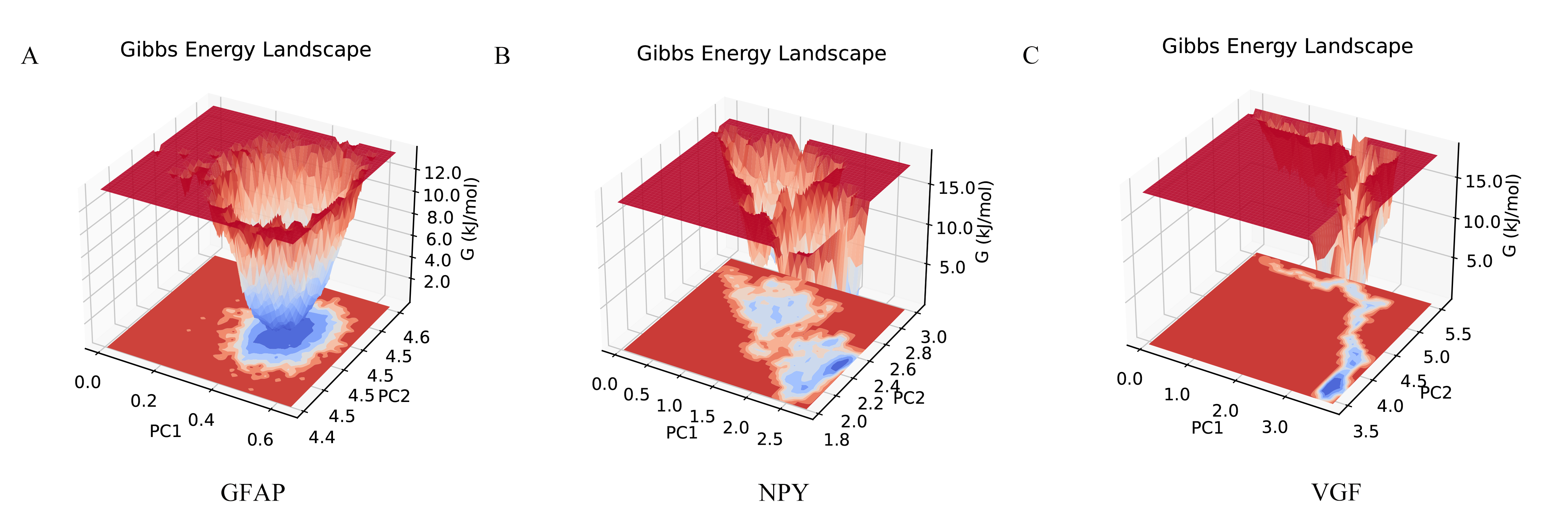


Figure S6. Molecular dynamics simulation results. A. Gibbs energy landscape of GFAP complexed with Geniposide; B. Gibbs energy landscape of NPY complexed with Geniposide; C. Gibbs energy landscape of VGF complexed with Geniposide; with the blue region indicating the lower energy conformation
